# Beyond the fold: experimentally traversing limit points in nonlinear structures

**DOI:** 10.1098/rspa.2019.0576

**Published:** 2020-01-29

**Authors:** Robin M. Neville, Rainer M. J. Groh, Alberto Pirrera, Mark Schenk

**Affiliations:** Bristol Composites Institute (ACCIS), University of Bristol, Bristol BS8 1TR, UK

**Keywords:** nonlinear structures, experimental path-following, structural stability, experimental mechanics

## Abstract

Recent years have seen a paradigm shift regarding the role of nonlinearities and elastic instabilities in engineering science and applied physics. Traditionally viewed as unwanted aberrations, when controlled to be reversible and well behaved, nonlinearity can enable novel functionalities, such as shape adaptation and energy harvesting. The analysis and design of novel structures that exploit nonlinearities and instabilities have, in part, been facilitated by advances in numerical continuation techniques. An experimental analogue of numerical continuation, on the other hand, has remained elusive. Traditional quasi-static experimental methods control the displacement or force at one or more load-introduction points over the test specimen. This approach fails at limit points in the control parameter, as the immediate equilibrium beyond limit points is statically unstable, causing the structure to snap to a different equilibrium. Here, we propose a quasi-static experimental path-following method that can continue along stable and unstable equilibria, and traverse limit points. In addition to controlling the displacement at the main load-introduction point, the technique relies on overall shape control of the structure using additional actuators and sensors. The proposed experimental method enables extended testing of the emerging class of structures that exploit nonlinearities and instabilities for novel functionality.

## 1. Introduction

In many human-made systems, nonlinearities and instabilities are viewed as unwanted aberrations. In the applied physics and engineering science communities, an alternative perspective has developed, whereby nonlinearities might be viewed as opportunities to enable novel functionality [1–3] or more efficient systems [4]. For example, buckling has been used for applications as diverse as energy harvesting [5,6], reversible shape adaptation [7,8], surface texturing [9], actuation [10], self-encapsulation [11], auxetic materials [12] and energy dissipation [13].

**Figure 1.**
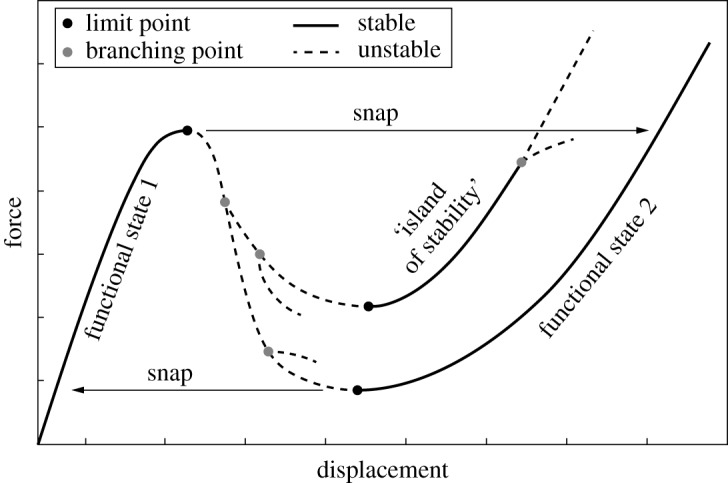
Schematic force–displacement response of a nonlinear structure that snaps between functional states, omitting an ‘island of stability’ with potentially additional features.

The physical mechanism that underpins many of these novel systems is the ubiquitous fold catastrophe, also known as the limit point or saddle–node bifurcation. In structural mechanics, the response of a load-bearing and deformable structure is generally described by equilibrium curves of applied (or resultant) force versus the resulting (or applied) displacement. When the structure reaches a limit point in the loading parameter (force or displacement), the stability of the structure changes—a previously stable equilibrium becomes unstable or vice versa. Consequently, a structure that is loaded by a slowly evolving, yet monotonously increasing, load snaps to another, often remote, equilibrium upon reaching a limit point [14]. This is because, although force(s) or displacement(s) at the loading point(s) are controlled, the rest of the structure is free to move dynamically. These snaps can toggle the structure between different functional states, but may also cause the structure to jump over additional stable segments of the force–displacement equilibrium manifold [15], as illustrated in figure 1. Because such ‘islands of stability’ are surrounded by unstable equilibria, they (i) are inaccessible to conventional experimental testing and (ii) cannot be exploited for additional functionality such as shape adaptation. Hence, new structural testing and control methods are needed to realize and exploit these shapes in a physical setting.

In this work, we focus on a relatively simple structure, a shallow arch loaded by a central point force, for which the mechanical behaviour is readily summarized in terms of curves relating the central transverse deflection to the resulting reaction force. For structures with more complex topologies and/or loading conditions, characterizing the nonlinear behaviour may require an extended parametrization of the results. However, for the proof-of-concept study considered herein, the lower order system is most instructive.

In recent years, a number of innovative experimental methods have been developed to explore the stability of nonlinear structures. Wiebe & Virgin [16] used hammer impacts to trigger the dynamic snap-through of shallow arches. The location of unstable equilibria was not computed directly, but inferred from the saddles traced by the arches’ dynamic phase-space trajectories. Virot *et al.* [17] used a ‘poker’ to laterally perturb an axially loaded cylinder to detect unstable edge states surrounding the stable pre-buckling equilibrium. An unstable equilibrium was found when the reaction force on the probe vanished, as this condition is equivalent to the ‘unprobed’ cylinder. Neville *et al.* [18] introduced the ability to both push and pull on ‘probes’ to control the deformed shape of a shallow arch, and was thus able to identify multiple unstable equilibria.

In order to experimentally path-follow along connected equilibria, i.e. an equilibrium path, a continuous control algorithm is needed. One promising approach to achieve this is to mirror the predictor–corrector schemes used for numerical continuation [19]. For example, van Iderstein & Wiebe [20] used additional control points to derive an experimental ‘tangent stiffness matrix’ to path-follow along an unstable equilibrium path of a post-buckled beam. However, the control algorithm did not converge at limit points where the tangent stiffness becomes singular. The capability of traversing limit points has, therefore, remained elusive in current experimental methods for quasi-static nonlinear structures.

In the related yet distinct field of structural dynamics, Sieber *et al.* [21,22] have developed the methodology known as control-based continuation (CBC). CBC allows dynamic continuation of periodic orbits through a fold, i.e. tracking of stable and unstable orbits, and thereby permits tracing of the full nonlinear backbone curve beyond a resonance peak. These methods rely on computing a Jacobian of the root-finding control signal. Ways to estimate the Jacobian in the naturally noisy environment of experiments are discussed by Schilder *et al.* [23] and Renson *et al.* [24,25].

The work presented herein establishes the same capability but in the field of statics, i.e. tracing of equilibria or stationary solutions. One key difference is that in dynamic systems the control inputs, excitation frequency and amplitude, can be varied independently. For static structural systems, the two control inputs—displacement and force—are linked through elasticity and need to be controlled indirectly via a third parameter: the structure’s shape. A second difference is that we focus on a Jacobian-free methodology that has the advantage of being less sensitive to noise but is more difficult to scale to larger dimensional systems.

In this paper, we embed the concept of *shape control* [18] in a continuous control algorithm that allows for experimental path-following of stable and unstable equilibrium branches, and the traversal of limit points. This will (i) enable the validation of structures that exploit nonlinearities for engineering applications; (ii) expand the design space for shape-adaptive structures by enabling access to ‘islands of stability’; and (iii) allow several longstanding numerical benchmarks found in the literature to be validated experimentally [26].

## 2. Shape control

**Figure 2.**
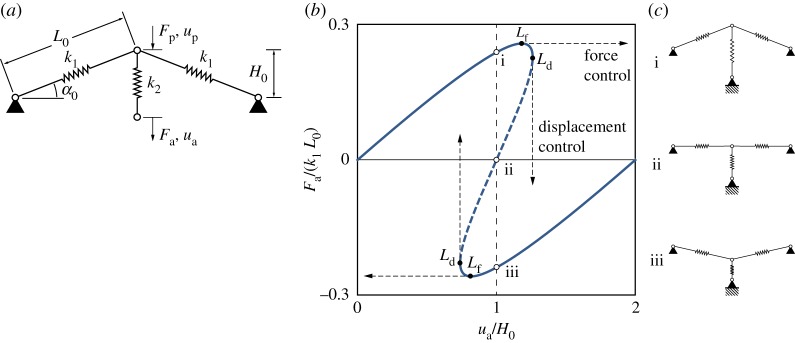
(*a*) von Mises truss geometry. The actuation point is at the bottom of the vertical spring; the probe point is at the apex of the truss. (*b*) The force–displacement curve for a von Mises truss with *k*_1_/*k*_2_ = 2 and *α*_0_ = 50^°^ has both displacement and force limit points. This causes the dashed section of the equilibrium curve to be unstable under either force or displacement control, and thus inaccessible to conventional quasi-static testing. The central section of the plot highlights three equilibria (points i, ii and iii) that correspond to three forces (*F*_a_) associated with one displacement (*u*_a_ = *H*_0_). (*c*) Each equilibrium i, ii and iii shown in (*b*) is associated with a unique truss shape. (Online version in colour.)

To illustrate the underlying concept of shape control and the associated control algorithm, we employ a simple structure that exhibits the salient features of nonlinear behaviour with limit points: a spring-loaded von Mises truss (figure 2). The truss features an arch-like arrangement of two inclined linear springs, with a third spring suspended from the apex. For a load applied to the bottom of the vertical spring, the force–displacement (*F*_a_ versus *u*_a_) response describes a general sigmoidal shape. The precise characteristics of this equilibrium curve are entirely described by the geometric arrangement (*α*_0_, *L*_0_) and stiffness ratio of the springs (*k*_1_/*k*_2_). For certain arrangements, the equilibrium curve features both force and displacement limit points (figure 2*b*).

The section of the equilibrium curve bounded by the two displacement limit points *L*_d_ (dashed segment in figure 2*b*) is experimentally inaccessible using conventional quasi-static testing techniques. This unstable segment of the equilibrium manifold acts as a repeller, whereas the two stable segments act as attractors. Hence, under displacement control (*u*_a_), the apex snaps up- or downwards upon reaching the unstable segment. To path-follow along the unstable equilibrium segment and to traverse both limit points, a method for simultaneously controlling the force and displacement at the loading point is needed. The experimental challenge is that force and displacement are inherently linked through elasticity: an applied force results in a displacement, and an applied displacement induces a reaction force.

For each unstable equilibrium within the dashed region of figure 2*b*, two further *stable* equilibria exist with the same actuation point displacement but different reaction force readings (see points i, ii and iii in figure 2*b*). These different shapes provide the key insight to decouple force and displacement at the actuation point; namely, by introducing a third control variable: the overall shape of the structure. For an applied displacement (*u*_a_), controlling the structure’s shape determines the corresponding reaction force (*F*_a_); conversely, for an applied force, the shape determines the displacement.

As shown in previous experimental work [18], the shape of the structure can be controlled by introducing additional control points. In the case of the von Mises truss, this is done by controlling the displacement of the apex (*u*_p_). Thus, the shape of the truss is uniquely determined by the relative positions of the apex and the actuation point. The purpose of these additional ‘probe’ points is twofold. First, for unstable equilibria, the probes provide the stabilization force required to resist dynamic snaps. Second, the probes can be used to select different equilibria that exist for a specific level of loading. Each unique equilibrium state of the unprobed structure must correspond to a zero reaction force reading at the probe points (*F*_p_ = 0). When this is the case—as far as the structure is concerned—the probes ‘do not exist’.

This concept of obtaining a zero force reading on the probes to pinpoint equilibria (stable and unstable) has two pertinent analogies in numerical methods: (i) the minimization of virtual work in response to a virtual displacement and (ii) the vanishing of the residual in Newton’s method. The principle of virtual work states that, of all possible kinematically admissible (virtual) deformations, the one that minimizes the total potential energy corresponds to the actual deformation. A powerful tool for solving the virtual work statement analytically or numerically is the collocation method (or alternatively the Galerkin method), whereby kinematically admissible shape functions are assumed and the residual at certain collocation points (or the total residual) is minimized. In precisely this fashion the probes are used to impose a subset of the kinematically admissible deformations (the ones that can be controlled by the probes), and the residual is then minimized at specific points (i.e. zero reaction force at the probe points). Similarly, most numerical frameworks used in structural mechanics—e.g. finite-difference or finite-element (FE) methods—divide the computational domain into discretization points (nodes). Some of these nodes are constrained from displacing (boundary conditions), others are loaded, and the rest are unloaded. As a reference load is applied, the unconstrained nodes displace, but, in general, there is a difference between the induced internal nodal forces and the applied external nodal forces. Hence, the structure is not in equilibrium. In Newton’s method, the structure is moved closer to an equilibrium state by applying the residual (the difference between internal and external nodal forces) as an additional force to *all* nodes. As a result, previously unloaded nodes of the structure are now loaded, thereby controlling the overall shape of the structure beyond the primary actuation point. An equilibrium state is found when the residual falls beneath a predefined threshold. The equivalent threshold in experimental shape control is the vanishing reaction force at the probe points.

In the following section, we define a simple control algorithm based on shape control that can path-follow stable and unstable equilibria and traverse limit points.

## 3. Step–scan control algorithm

**Figure 3.**
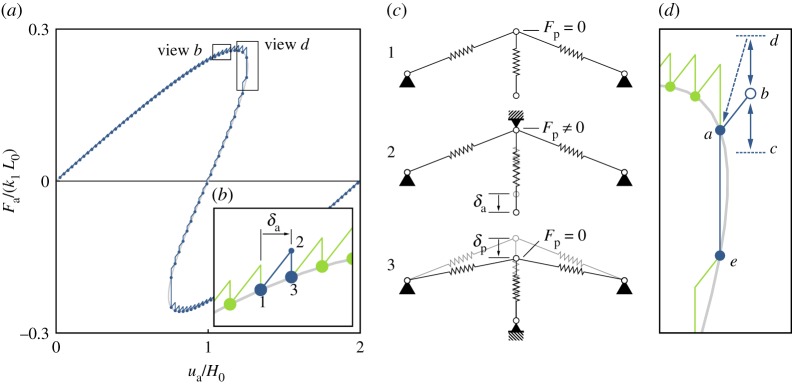
(*a*) Schematic of the experimental path-following algorithm (blue steps) superimposed on the equilibrium curve (grey). (*b*) Detail view of the stepping procedure described by algorithm ?? in appendix A. Starting at point 1 on the equilibrium curve, the probe is fixed and the actuation point is moved by increment *δ*_a_, which generates a non-zero probe force (point 2). The probe is then moved until a zero probe force reading is found (point 3). The shapes associated with these points are shown conceptually in (*c*). (*d*) Detail view of the limit-point tracing logic described by algorithm ?? in appendix A. Starting at point *a*, we step by *δ*_a_ to point *b*. The probe is then moved to search for zero probe force, but there are no solutions within bounds *c* and *d*. The algorithm returns to the known equilibrium point *a*, and moves the probe until the next solution is found at point *e*. (Online version in colour.)

To implement a path-following algorithm, a combination of controlling the displacement at the actuation point, *u*_a_, and scanning for equilibria using the probe point, *u*_p_, is required. The simplest implementation of such an algorithm is here referred to as the *step–scan* method and is demonstrated schematically on the von Mises truss in figure 3. First, the method involves a finite increment, or *step*, *δ*_a_, of the actuation point at constant probe displacement, *δ*_p_ = 0. This step (moving from 1 to 2 in figure 3*b*) induces a non-zero reaction force at the probe point. Next, the probe point is moved by *δ*_p_ until the probe reaction force reads zero (2 to 3 in figure 3*b*), i.e. the probe *scans* for an equilibrium. This procedure is formally described by algorithm ?? in appendix A. This basic step–scan algorithm is used to progress along both statically stable and unstable equilibrium paths, as shown graphically in figure 3*b* by the saw-tooth-shaped segments and schematically in figure 3*c* by the deformed shapes. As long as the actuation point increment, *δ*_a_, is sufficiently small, the probe-scanning step (moving *δ*_p_) quickly encounters a zero reaction force reading, *F*_p_ = 0. However, this algorithm fails to converge at a displacement limit point because the control point increment, *δ*_a_, takes the system into a region where the probe scanning step does not intersect an equilibrium solution (figure 3*d*).

To overcome this limitation and find a new equilibrium past the limit point, the proposed control system first probe-scans in one direction and, upon failing to identify a zero reaction force reading within a preset bound (point *c*), inverts to scan in the opposite direction. If a zero probe reaction force is also not found in this scanning direction (point *d*), the control system returns the structure to its previously identified equilibrium (point *a*), followed by a further probe scan in the original direction to find the next equilibrium (point *e*). This procedure is formally described by algorithm ?? in appendix A. Throughout this procedure, the controlling action of the probes prevents snapping away from unstable equilibria.

Although not as sophisticated as numerical continuation methods, the simplicity of the proposed method provides a robustness which is beneficial when considering the noise and other imperfections that are present in an experiment. Most importantly, the algorithm is capable of dealing with the displacement reversal at limit points, allowing it to path-follow from the stable portion of the equilibrium curve onto the unstable one without dynamic snapping. In the following section, we demonstrate the successful implementation of the step–scan algorithm on a symmetric shallow circular arch loaded vertically at its midpoint (the actuation point). To the best of our knowledge, this is the first time limit-point traversal in a quasi-static setting has been demonstrated experimentally.

## 4. Experimental implementation

**Figure 4.**
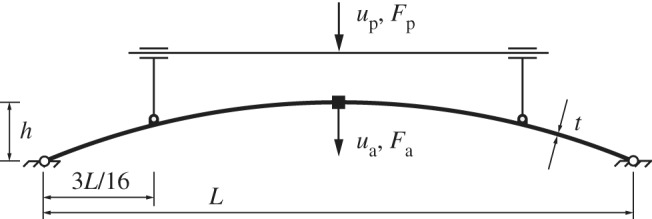
The symmetric shallow arch studied in this work with midpoint displacement *u*_a_; midpoint rotation is prevented to enforce symmetry. The additional ‘probes’ control the deformed shape by imposing vertical displacement *u*_p_.

A transversely loaded shallow circular arch was selected for the experiments, as it demonstrated nonlinear behaviour with multiple limit points; the geometry is shown in figure 4. The load was applied at the midpoint. Symmetry-breaking bifurcations, and the consequent branching off the main equilibrium manifold, were avoided by preventing midpoint rotation. This symmetry condition is imposed herein as the natural behaviour of the arch is to transition between the original and everted states via an asymmetric mode that is stable under displacement control. Hence, the asymmetric mode does not require stabilization via the experimental path-following methods described herein. Second, the equilibrium manifold corresponding to the asymmetric deformation mode does not feature any limit points. Hence, to illustrate limit-point traversal and path-following along unstable paths, we enforce left–right symmetry by restricting midspan rotations and using linked probe pairs.

The arch shape was controlled using two linked (again, to maintain symmetry) probe points at 3*L*/16 from the supports, which imposed vertical displacement but allowed free rotation and horizontal translation. The arch specimens used in this work were laser cut from acrylic sheets. Young’s modulus and Poisson’s ratio of the material are *E* = 3200 MPa and *ν* = 0.38. The specimens had nominal dimensions *L* = 205 mm, *h* = 20 mm, *t* = 1.5 mm as per figure 4, and depth *d* = 5 mm (into the page).

**Figure 5.**
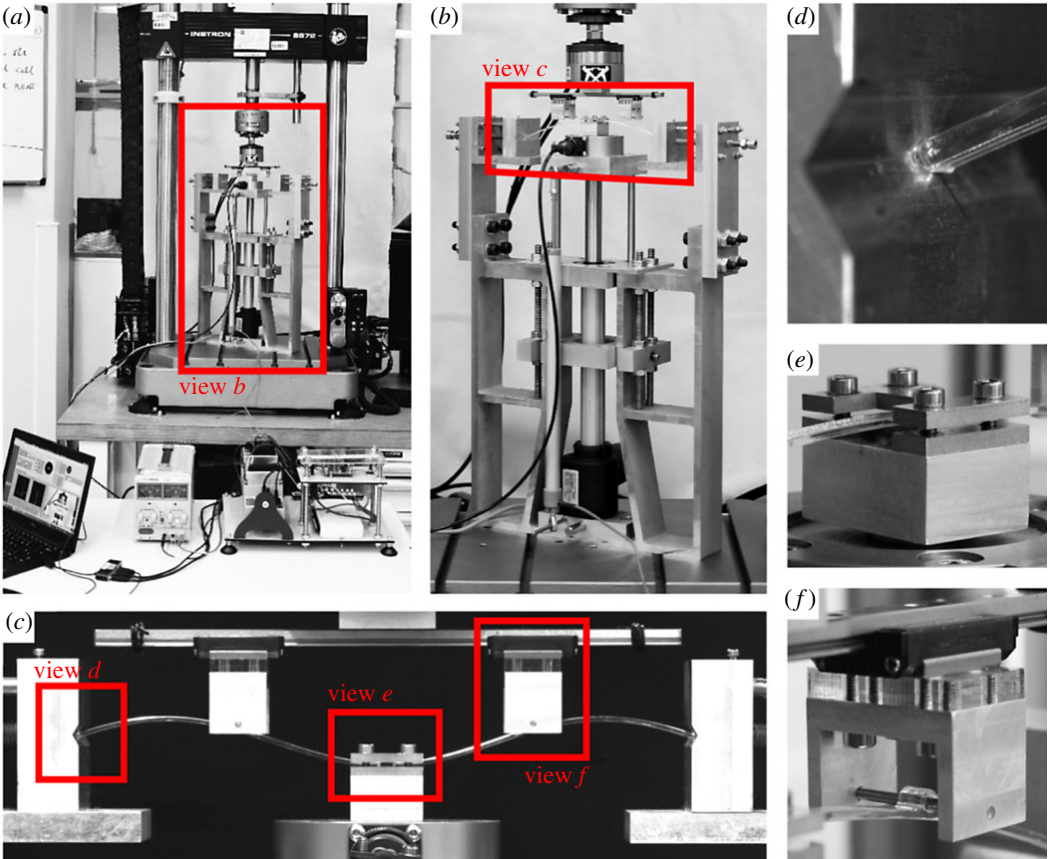
Experimental set-up. (*a*) LabVIEW laptop and electronics in front of the Instron. (*b*) The test frame with linear transducer, actuator and load cell attached to the base of the Instron. (*c*) The deformed arch specimen with central actuation point and probes attached. (*d*) The end of the arch resting in the wedge block. (*e*) The ‘I’-shaped plates clamping the arch midpoint. (*f* ) The pins which attach the probes to the arch. (Online version in colour.)

The experimental set-up is shown in figure 5. The experiment performed in this work requires two independent displacement-controlled inputs—one for the actuation point and one for the probes. A purpose-built experimental set-up was used to support the arch at its edges, to control the actuation point position and to measure the actuation point force. The frame was fixed to the base of an Instron universal test machine, which was used to control the probe position and measure the probe force. For further details regarding the components used, see Neville *et al.* [27]. The path-following algorithm was implemented in LabVIEW, which controlled the displacements of the midpoint and probes and recorded the corresponding reaction force readings.

The pinned end boundary conditions for the arches were implemented using wedge-shaped blocks (figure 5*d*), which allowed the arch ends to rotate while restraining translations. The midpoint of the arch was clamped between two plates (figure 5*e*); the width of the clamped area was 5 mm. The probes were attached to the arch by 2 mm diameter steel pins, which passed through loops in the arch (figure 5*f* ). A linear rail allowed free movement of the probes in the horizontal direction, while enforcing equal vertical position.

## 5. Results

**Figure 6.**
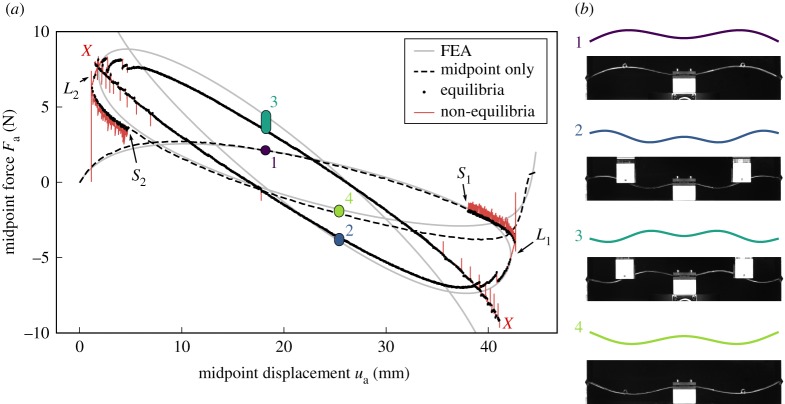
(*a*) Comparison of the load–displacement equilibrium plots obtained by the step–scan algorithm and a nonlinear FE prediction. The FE simulations used measured (rather than nominal) arch dimensions: *L* = 204.3 mm, *h* = 21.3 mm, *t* = 1.56 mm and *d* = 5.0 mm. (*b*) Mode shapes from the FE model at points 1–4 and the matching experimental mode shapes. (Online version in colour.)

The experiment was performed in two stages. First, a run was performed with displacement control at the arch midpoint. The midpoint displacement, *u*_a_, was increased until the fully inverted arch shape was obtained; then *u*_a_ was decreased to revert the structure back into its original unloaded position. Under this midpoint-only displacement control, the arch snapped at the two displacement limit points, *L*_1_ and *L*_2_, producing the dashed load–displacement curve in figure 6*a*.

After obtaining the conventional force–displacement curve, the arch was taken to a position just before one of the limit points (*S*_1_ and *S*_2_ in figure 6*a*). The probes were attached to the arch, and the path-following algorithm was initiated. This experiment was performed twice in order to traverse both *L*_1_ (from *S*_1_) and *L*_2_ (from *S*_2_). The following algorithm parameters were selected: probe force residual |*F*_p_| < 0.1 N as the equilibrium threshold, and actuation point step size *δ*_a_ = 0.1 mm. A video of the experimental path-following is provided in the electronic supplementary material.

Figure 6 shows the experimental results compared with the FE analysis (FEA) predictions. The algorithm’s output is shown in black and red, with black denoting all converged equilibria (probe force |*F*_p_| < 0.1 N) and red denoting out-of-equilibrium states. The curve from a nonlinear FE prediction is shown in grey. The experimental curves are qualitatively similar to the FE curves, with some small quantitative differences. The spikes in the red non-equilibrium data at the limit points were caused by the probes unsuccessfully scanning for equilibria before reversing direction and re-encountering the equilibrium curve, as described in the algorithm of figure 3. The arch shapes observed on the two unstable portions of the equilibrium curve match those predicted by the FEA (figure 6*b*) in terms of both number and position of the half-waves.

As shown in figure 6*a*, the experimental path-following framework is able to traverse the two limit points *L*_1_ and *L*_2_ and follow the unstable equilibrium path, but cannot go beyond the next set of limit points (two red *X*-marks) where dynamic snaps adjacent to the probes occur. This second set of limit points separates deformation-mode shapes with five and seven half-waves. The experimental path-following set-up does not possess sufficient deformation fidelity—i.e. number and location of probes—to control the higher order deformation mode shapes, and this results in a loss of ‘control authority’. The *a priori* selection of the number and location of probes is an important factor in successful experimental path-following, as these determine the equilibria that can be identified.

**Figure 7.**
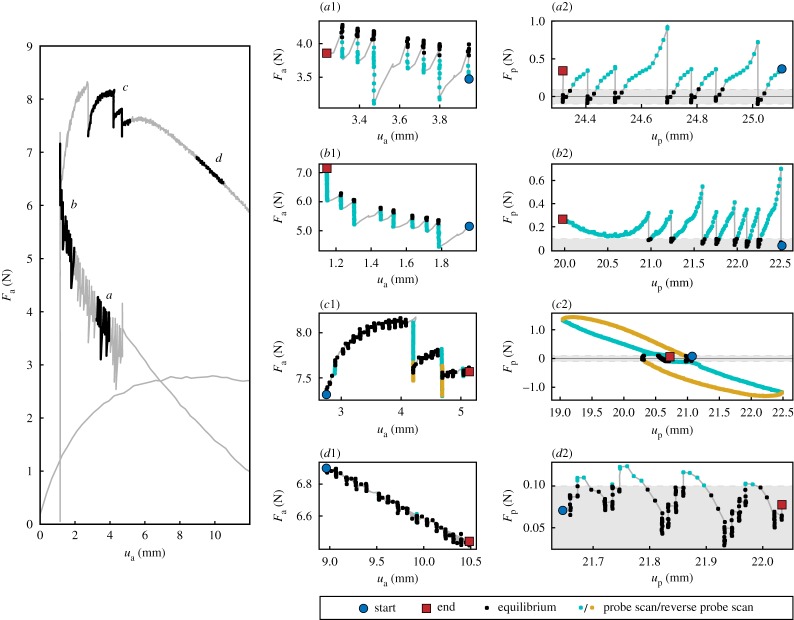
Details of step–scan algorithm results near limit point *L*_2_, with areas of interest (*a*–*d*) highlighted. *a* and *b* are stable segments, and *c* and *d* are unstable segments of the equilibrium path. The subplots on the right show detailed views of the data in each area of interest. The middle column of subplots (*a*1–*d*1) are zoomed-in views of the equilibrium curve (*u*_a_ versus *F*_a_ space). The right column of subplots (*a*2–*d*2) show the *F*_p_ versus *u*_p_ data collected from the probes. The colours on subplots *a*1*–*d2 indicate different algorithm states: black dots are equilibria, cyan dots are part of a probe scan and yellow dots are part of a reverse probe scan. The grey shaded area indicates the equilibrium criterion range |*F*_p_| < 0.1 N. The blue dot and red square indicate, respectively, the first and last data point logged in the area of interest during the testing sequence. (Online version in colour.)

Figure 7 details the measurements near limit point *L*_2_ for both the actuation point and the probes. Subplots *a*1 and *a*2 show a stable segment of the equilibrium path away from the limit point. Subplot *a*1 is a zoomed-in version in *u*_a_ versus *F*_a_ space, whereas subplot *a*2 shows the corresponding probe data in *F*_p_ versus *u*_p_ space. The tolerance of convergence |*F*_p_| < 0.1 N is marked by a grey band. The ‘sawtooth’ pattern generated by the algorithm is clearly visible. The gradient of the probe scans on subplot *a*2 is positive, indicating stable equilibria. Subplots *b*1 and *b*2 show the stable area close to limit point *L*_2_. Starting at the blue circle in subplot *b*2, the gradients of the probe scans become increasingly shallow as the algorithm approaches the limit point, until the midpoint steps past the limit point and the algorithm cannot find an equilibrium (*F*_p_ remains greater than 0.1 N). Eventually, the gradient becomes negative near the red square.

Subplots *c*1 and *c*2 show ‘jumps’ in the equilibrium curve on the unstable region near the limit point. Because of the nature of the equilibrium criterion (|*F*_p_| < 0.1 N) there will be a range of probe forces *F*_p_ (and hence a range of probe positions *u*_p_) which satisfy the equilibrium condition. Consequently, the algorithm does not measure the precise equilibrium curve, but rather a bounded area around the equilibrium curve within the specified tolerance on *F*_p_. On most parts of the equilibrium curve, the probe force gradient (d*F*_p_/d*u*_p_) is sufficiently steep to ensure narrow bounds, and this guarantees a close approximation to the actual equilibrium curve. As seen in subplot *b*2, the probe force gradient is much shallower near the limit points, which results in wider bounds. The ‘jumps’ are caused by the algorithm performing multiple consecutive step–scan iterations inside the equilibrium tolerance band (|*F*_p_| < 0.1 N). The algorithm may exit the equilibrium tolerance band, and the probes scan in a direction that moves the solution away from equilibrium. One example of this phenomenon is shown in subplot *c*2; the first part of the probe scan (cyan dots) increases the probe force. As the algorithm does not find an equilibrium, it reverses the direction of the probe scan. This reversed scan (yellow dots) re-encounters the equilibrium area for a different probe displacement *u*_p_ than it originally exited. This hysteresis is thought to be due to stick-slip behaviour at the boundary conditions, and could be resolved through improved experimental design.

Finally, subplots *d*1 and *d*2 show the unstable equilibrium segment further away from the limit point. The unstable nature of the equilibria is reflected by the negative gradient of the *F*_p_ versus *u*_p_ subplot in *d*2. The probe scan direction finds equilibria within the equilibrium bounds, avoiding the hysteresis due to reversal of the scanning direction discussed previously.

We conclude this section by commenting on the robustness of the arch response—and therefore of the algorithm—to aleatoric uncertainties as well as small geometric and constitutive changes. It is well known that the transverse force versus transverse displacement behaviour of a circular arch can be expressed using the non-dimensional transverse displacement *u*_a_/*R* and non-dimensional load *F*_a_ *R*^2^Θ/*EI*, where *R* is the radius of curvature, Θ is half of the included angle, *E* is Young’s modulus and *I* is the second moment of area. Hence, the arch is most sensitive to changes in the thickness *t* (through the second moment of area *I*) and radius *R* of the arch. In addition to geometric and constitutive uncertainty, experimental noise and measurement uncertainty affect the results. Previous numerical studies investigated the effects of sensor noise [28]. The observations made therein—that the sensitivity to measurement uncertainty is greatest around the limit points and smallest around the predominantly linear portions of the curve—are corroborated in our experimental work. In the presented algorithm, the primary parameter was chosen to be *u*_a_, such that steps in *u*_a_ always remain in the outer loop of the algorithm and scans in *u*_p_ in the inner loop. Potential improvements in results could be achieved by switching to *u*_p_ in the outer loop in the vicinity of the limit point in *u*_a_, and vice versa. This would remove singularities in the fundamental loading parameter, either *u*_a_ or *u*_p_, allowing for more robust traversal of either limit point and a minimization of the susceptibility to noise. Two additional factors during testing are creep/relaxation and plastic deformations as the acrylic arch is forced into the higher order mode shapes beyond the limit points. Relaxation occurs as the step sizes are relatively small, such that the arch remains in a highly strained configuration for long periods of time. Furthermore, the high strain values experienced in the high-order mode shapes may also plastically deform the specimens. Despite these considerations and the fact that the acrylic specimens were manufactured using a laser cutting process with low dimensional fidelity, it was found that the mechanical response of individual specimens was surprisingly repeatable.

## 6. Outlook

The notion of ‘control authority’ suggests that, with increasing structural complexity (e.g. from arch to shell), the number of probe points to control the structure increases correspondingly. A practical experimental path-following set-up under these circumstances requires a more sophisticated control algorithm. Ideally, one that moves a set of control points concertedly based on the non-zero reaction force readings at all probe points, i.e. an experimental analogue to numerical continuation. Indeed, the general control signal *F*_p_(*u*_a_, *u*_p_) = 0 can also be solved by adapting one of the many available numerical continuation solvers into a LabVIEW environment. This would require the computation of the Jacobian of *F*_p_ using a finite-difference approximation by slightly perturbing *u*_a_ and *u*_p_. As demonstrated by van Iderstein & Wiebe [20], an experimental tangential stiffness matrix can be computed by perturbing a single probe and recording the change in reaction force at all control points. By repeating this procedure for all probes sequentially, a finite-difference tangential stiffness matrix can be assembled. In this manner, the testing method can not only be scaled up to more complex structures, but the algorithm also departs from the simple step–scan approach to a more refined experimental path-following method. The advantage of this refined approach is that a predictor–corrector scheme can be implemented, whereby both *u*_a_ and *u*_p_ are controlled during the predictive step away from an equilibrium and during the ensuing corrective steps. The disadvantage of the approach is that the computation of the Jacobian is exceedingly sensitive to noise close to the limit point, such that alterations to the typical numerical continuation algorithms are necessary for a successful implementation in an experimental setting.

The objective of the present work is to show that a Jacobian-free method is also possible that relies on *u*_a_ only for the predictor and *u*_p_ only for the corrector, and is therefore more robust to experimental noise. A variant of the experimental path-following technique based on shape control that relies on the Jacobian has also been successfully implemented by the present authors, but is beyond the scope of the present work. In conclusion, the simplicity of the step–scan algorithm means it is less sensitive to experimental noise, and can therefore reliably traverse limit points. In fact, simulations have shown pronounced sensitivity of the finite-difference tangential stiffness matrix close to the limit points, hindering their reliable traversal. Nonetheless, with the development of appropriate control algorithms, the traversal of limit points is expected to be achievable [29].

## 7. Conclusion

This paper presents a new testing method for nonlinear structures. The proposed quasi-static experimental path-following method can continue along stable and unstable equilibrium branches, and traverse limit points in a structure’s force–displacement response—it is able to explore beyond the fold. To the best of our knowledge, this is the first time this capability has been demonstrated experimentally. The methodology relies on the concept of shape control, whereby additional control points are introduced across the structure to stabilize unstable equilibria and prevent snapping at limit points. A simple step–scan algorithm is implemented experimentally to traverse two limit points of a transversely loaded shallow arch.

Fundamentally, we have demonstrated a direct mapping of familiar numerical quantities to the experimental domain: the introduction of probe points enables the calculation of an experimental tangential stiffness matrix and the residual force vector, required for Newton’s method. This means that well-understood numerical algorithms can be adapted for experimental path-following.

We expect that these experimental path-following methods will enhance engineers’ and scientists’ capabilities to test and validate buckling-driven multi-functional structures. Furthermore, they will enable novel functionality by providing access to equilibrium states that are currently inaccessible.

## Supplementary Material

Video of Experimental Path-Following

## Data accessibility

All data underlying this publication can be downloaded from the data repository of the University of Bristol at https://doi.org/10.5523/bris.1vw1mrp7hm84j2iw9o4m1whkcz.
